# Effectiveness of interventions for pain relief in hysterosalpingographyAnetwork meta-analysis and systematic review

**DOI:** 10.12669/pjms.334.13065

**Published:** 2017

**Authors:** Xin Guo, Zongjian Tan

**Affiliations:** 1 XinGuo, Department of Reproductive Center, Guizhou Provincial People’s Hospital, Guiyang, Guizhou, China; 2 Zongjian Tan, Department of Reproductive Center, Guizhou Provincial People’s Hospital, Guiyang, Guizhou, China

**Keywords:** Hysterosalpingography, Pain relief, Network Meta-analysis

## Abstract

**Objective:**

We aimed to evaluate the effectiveness of placebo, oral opioid analgesic (OOA), intravenous opioid analgesic (IOA), non-opioid analgesic (NOA), topical anesthetic (TA) and locally injected anesthetic (LIA) for pain relief duringhysterosalpingography (HSG) using a Bayesian network meta-analysis of data from randomized controlled trials.

**Methods:**

PUBMED, EMBASE, and CENTRAL search engines were used to search and identify clinical trials that evaluated interventions for pain relief in HSG. Methodological studies quality was assessed by the Cochrane Collaboration tool for assessing risk of bias.

**Result:**

Sixteen trials involving 1263 participants were included in this study. IOA got excess but not statistically significant lower visual analogue score (VAS) pain score during HSG or more than 30 minutes after HSG compared with the other groups. OOA resulted in excess but not statistically significant higher VAS pain score during HSG compared with the other groups except placebo group. According to SUCRA regarding the lower VAS pain score during HSG, the treatments rank was the following: IOA, TA, NOA, LIA, OOA and placebo; as regard lower VAS pain score at 30 minutes or more after HSG, the treatments rank was the following: IOA, LIA, OOA, TA, NOA and placebo.

**Conclusion:**

This new Bayesian data network meta-analysis from randomized controlled trials demonstrated that IOA resulted in the highest probability to reduce the pain during HSG or at 30 minutes or more after HSG among the six interventions considered.

## INTRODUCTION

Approximately 14% of infertilitycases are due to fallopian tubes abnormalities.^1^ Therefore, it is crucial to assess fallopian tubes patency during the diagnosis of infertility.^2^ Hysterosalpingography (HSG) is considered as the gold standard in the assessment of tubal patency^3^ and it is also recommended by the National Institute for Health and Care Excellence (NICE) guidelines for tubal occlusion evaluation in the absence of co-morbidities.^4^

Although HSG is particularly useful in the diagnosis of uterine abnormalities and intrinsic tubal disease,^5^,^6^ unfortunately, up to 72% of women complain of pain with this test.^7^ Pain can be caused by cervical instrumentation, uterine distension, and peritoneal irritation due to contrast spill into the peritoneal cavity.^8^ A variety of pain relievers, such as oral opioid analgesic (OOA), intravenous opioid analgesic (IOA), non-opioid analgesic (NOA), topical anesthetic (TA) and locally injected anesthetic (LIA) have been used to reduce pain during and after HSG. The debate among gynecologists and obstetricians regarding the best medication is as yet unresolved since 1980s. Previous pairwise meta-analysis^8^ could not get hierarchies of these treatments because some treatments had not been compared one by one.

We aimed to compare the effectiveness of five treatments (OOA, IOA, NOA, TA, LIA) for pain relief in HSG. Our intention was to provide hierarchies of the comparative visual analogue score (VAS) during HSG and VAS more than 30 minutes after HSG.

## METHODS

### Eligibility criteria and literature search

This study was performed in accordance with the Preferred Reporting Items for Systematic Reviews and Meta-Analyses (PRISMA) statement.^9^ Cochrane Register of Controlled Trials (CENTRAL, Aug 2016), PubMed (Jan 1980 to Aug 2016), and EMBASE (1980 to Aug 2016) databases were used to identify all studies analyzing the effectiveness of pain relievers due to HSG. Keywords and MeSH terms including “hysterosalpingography”, “pain”, “treatment”, “therapy” and “randomized controlled trial” were used in our search strategy.

The inclusion criteria were the following: (1) target population:Women attending for HSG to evaluate tubal patency; (2) intervention: OOA, IOA, NOA, TA, LIA; (3) methodological criteria: randomized controlled trials. The exclusion criteria were the following: (1) target population:women with pelvic inflammatory disease or any other cause of chronic pelvic pain and history of chronic narcotic or benzodiazepine use; (2) methodological criteria: case control study, case reports and cohort studies. The study selection was conducted by two independent reviewers. Any disagreement between the two reviewers was resolved by discussion.

### Outcome assessment

VAS pain score during and at 30 minutes or more after HSG.

### Data extraction and quality assessments

Random sequence generation, allocation concealment, blinding, selective reporting and incomplete outcome data were gathered from all randomized controlled trials. Study type, country, sample size and interventions data were also collected from each trial. In addition, the following clinical data were extracted if available: VAS during HSG and VAS at 30 minutes or more after HSG.

Cochrane Collaboration tool for assessing risk of bias was used to assess the quality of randomized controlled trials, using the following criteria: (1) randomization sequence generation: assessment for selection bias; (2) allocation concealment: assessment of selection bias; (3) level of blinding (blinding of participants and blinding of outcome assessment): assessment for performance bias and detection bias; (4) incomplete outcome data: assessment for attrition bias; and (5) selective reporting: assessment for reporting bias.^10^

### Data synthesis and analysis

Either VAS during HSG or VAS at 30 minutes or more after HSG was a continuous outcome, so we calculated the mean differences (MD) for each study with comparable outcome measures using a 95% confidence interval (CI). Two researchers extracted the data independently according to the prespecified selection criteria. Disagreements were resolved by discussion.

Conventional pairwise meta-analyses for all outcomes and comparisons were performed using a random-effects model by STATA (version 12.0, Stata Corp, College Station, TX). The pooled estimates of SMDs and 95% CI of two outcomes (VAS during HSG and VAS at 30 minutes or more after HSG) were shown. Network meta-analysis combining direct and indirect evidence within a Bayesian framework was implemented using the statistical software WinBUGS (version 1.4.3) from the packages of Markov Chain Monte Carlo (MCMC). The models, codes, software WinBUGS used in this study are freely available online.^11^ Surface under the cumulative ranking curve (SUCRA) probabilities was performed to rank the five interventions for treating displaced proximal humeral fracture:^12^ SUCRA is a proportion, expressed as a percentage of the efficacy of an intervention on the outcome that would be ranked first without uncertainty, which equals 100% when the treatment is certain to be the best and 0% when it is certain to be the worst.^12^ Thus, the larger the SUCRA value, the better the rank of the treatment.

Higgins model to assess the inconsistencies of this network meta-analysis was also used. Significance levels of less than 0.05 for the Higgins model test were considered as evidence of inconsistency. The sensitivity analysis was performed by changing the effect model (random-effect to fixed-effect model) and comparing the results of different effect model.

## RESULTS

### Study selection

**Fig. 1:**
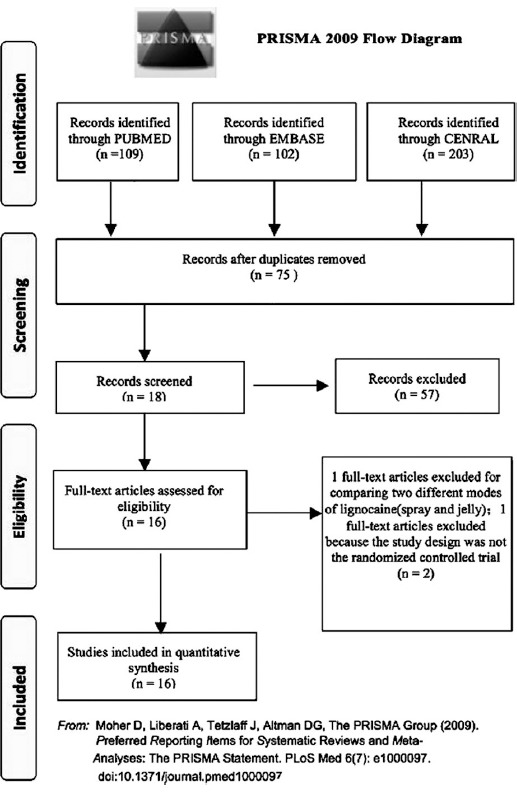
Flow chart of selection of studies for inclusion in meta- analysis.

Fig. 1 shows the study selection process according to the PRISMA statement. This search strategy retrieved a total of 414 studies; 203 studies were found using CENTRAL, 109 studies using PUBMED and 102 studies using EMBASE. Titles and abstracts of these references were examined by two reviewers, and 18 studies were selected for further analysis. Two studies were excluded: one because was related to the comparison of two different modes of lignocaine (spray and jelly)^13^ and another one was not a randomized controlled trial.^14^ Sixteen randomized controlled trials^3^,^6^,^15^-^18^,^22^,^23^,^28^ were considered as primary relevant studies and included in this network meta-analysis.

### Study characteristics and risk of bias in the included studies

Table-I provides a summary of the studies included in this review. A total of 1263 participants were considered, with sample size ranging from 20 to 128. All the 16 studies were comparing one treatment to another. These studies were published between 1985 and 2016. Sixteen studies reported VAS during HSG as an outcome and eleven studies evaluated VAS pain score at 30 minutes or more after HSG as an outcome.

**Table-I:** Characteristics of included studies comparing different treatments for pain relief in hysterosalpingography.

*Study*	*Country*	*Interventions*	*Sample size*	*Study design*	*For analysis*
Gupta 2008	India	TA vs NOA	50/50	RCT	VAS pain score more than 30 minutes after HSG
Cengiz 2006	Turkey	IOA vs PLB	32/30	RCT	VAS pain score during HSG, VAS pain score more than 30 minutes after HSG
Chauhan 2013	India	LIA vs PLB	50/50	RCT	VAS pain score during HSG
Costello 2002	Australia	TA vs PLB	55/55	RCT	VAS pain score during HSG
Elson 2000	UK	NOA vs PLB	39/49	RCT	VAS pain score during HSG, VAS pain score more than 30 minutes after HSG
Frishman 2004	USA	TA vs PLB	63/64	RCT	VAS pain score during HSG
Jacobs 1991	USA	TA vs LIA	10/10	RCT	VAS pain score during HSG, VAS pain score more than 30 minutes after HSG
Kafali 2003	Turkey	TA vs PLB	41/45	RCT	VAS pain score during HSG, VAS pain score more than 30 minutes after HSG
Kalantari 2014	Iran	TA vs PLB	40/40	RCT	VAS pain score during HSG, VAS pain score more than 30 minutes after HSG
Karaman 2016	Turkey	NOA vs PLB	42/40	RCT	VAS pain score during HSG, VAS pain score more than 30 minutes after HSG
Karasahin 2009	Turkey	TA vs PLB	27/14	RCT	VAS pain score during HSG
Liberty 2007	Israel	TA vs PLB	41/37	RCT	VAS pain score during HSG
Owens 1985a	USA	NOA vs PLB	15/7	RCT	VAS pain score during HSG, VAS pain score more than 30 minutes after HSG
Owens 1985b	USA	NOA vs PLB	15/8	RCT	VAS pain score during HSG, VAS pain score more than 30 minutes after HSG
Peters 1996	Netherlands	OOA vs NOA	42/49	RCT	VAS pain score during HSG, VAS pain score more than 30 minutes after HSG
Stoop 2010	Belgium	OOA vs PLB	67/61	RCT	VAS pain score during HSG, VAS pain score more than 30 minutes after HSG
Unlu 2015	Turkey	LIA vs PLB	18/7	RCT	VAS pain score during HSG

OOA: oral opioid analgesic; IOA: intravenous opioid analgesic; NOA: non-opioid analgesic; TA: topical anaesthetic; LIA: locally injected anaesthetic; HSG: hysterosalpingography; VAS: visual analogue score

Of the sixteen randomized controlled trials analyzed, Cochrane Collaboration tool indicated that ten trials^15^-^19^,^22^,^24^-^27^ used an adequate randomization and three trials^3^,^18^,^22^ used an adequate allocation concealment. Five studies^3^,^17^,^18^,^22^,^24^ reported adequate blinding and seven studies^3^,^16^,^19^,^23^,^24^,^26^,^27^ were free of selective reporting. Ten trials^3^,^16^-^18^,^20^-^23^,^25^,^27^ reported incomplete outcome data (Table-II).

**Table-II:** Quality assessment of randomized controlled trials comparing different interventions for pain relief in hysterosalpingography using the cochrane collaboration tool for assessing risk of bias.

*Author group*	*Adequate randomization*	*Adequate allocation concealment*	*Adequate blinding*	*Incomplete outcome data reporting*	*Free of selecting report*	*Other bias*
Gupta 2008	Y	Unclear	N	N	Y	Y
Cengiz 2006	Y	Unclear	Unclear	Unclear	Unclear	Y
Chauhan 2013	Y	Unclear	N	Y	Y	Y
Costello 2001	Y	Unclear	Y	Y	Unclear	Unclear
Elson 2000	Unclear	Unclear	Unclear	N	Unclear	Y
Frishman 2004	Y	Y	Y	Y	Unclear	Unclear
Jacobs 1991	Unclear	Unclear	Unclear	Y	Unclear	Unclear
Kafali 2003	Unclear	Unclear	Unclear	Y	Unclear	Y
Kalantari 2014	Y	Y	Y	Y	Unclear	Y
Karaman 2016	Unclear	Y	Y	Y	Y	Unclear
Karasahin 2009	Unclear	Unclear	N	Y	Y	Y
Liberty 2007	Y	Unclear	Y	Unclear	Y	Y
Owens 1985	Y	Unclear	Unclear	Y	Unclear	Y
Peters 1996	Y	Unclear	Unclear	Unclear	Y	Unclear
Stoop 2010	Y	Unclear	Unclear	Y	Y	Y
Unlu 2015	Unclear	Unclear	N	Unclear	N	Unclear

Y, Low risk of bias; N, High risk of bias; Unclear, Unclear risk of bias.

### VAS during HSG

**Fig. 2:**
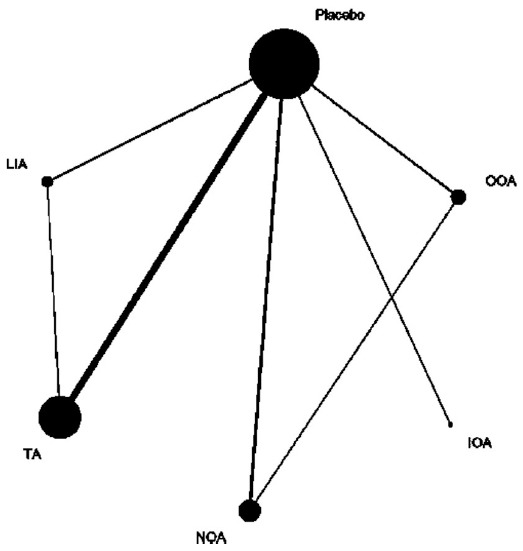
Network of treatment comparisons for VAS during HSG. The size of the node corresponds to the total sample size of treatments. Directly comparable treatments are linked with a line, the thickness of which represents the number of trials that were compared. OOA: oral opioid analgesic; IOA: intravenous opioid analgesic; NOA: non-opioid analgesic; TA: topical anaesthetic; LIA: locally injected anaesthetic; HSG: hysterosalpingography; VAS: visual analogue score.

All the 16 trials were included in the network meta-analysis to evaluate this outcome. The VAS comparisons network during HSG is shown in Fig. 2. Table-III provides a hierarchy of effects based on VAS score during HSG. The direct and indirect comparisons indicated that IOA got excess but not statistically significant lower VAS pain score during HSG compared with the other groups. The same comparisons also indicated that OOA also resulted in excess but not statistically significant higher VAS pain score during HSG compared with the other groups except placebo group. Based on SUCRA, IOA (0.9928) ranked as first, followed by TA (0.6826), NOA (0.4806), LIA (0.3550), OOA (0.3368) and placebo (0.1522).

**Table-III:** Results for VAS pain score during HSG, from network meta-analysis (lower diagonal part) and pairwise meta-analysis (upper diagonal part).

Placebo	0.91(-0.06-1.88)	3.53(2.77-4.29)	0.39(-0.12-0.92)	0.69(0.17-1.2)	1.31(1.07-1.55)
0.29(-1.27-1.86)	OOA	N/A	1.10(-0.26-2.46)	N/A	N/A
3.54(1.43-5.57)	3.25(0.7-5.79)	IOA	N/A	N/A	N/A
0.55(-0.4-1.51)	0.26(-1.25-1.86)	-2.99(-5.22--0.61)	NOA	N/A	N/A
0.97(0.24-1.76)	0.68(-1.04-2.47)	-2.57(-4.72--0.48)	0.42(-0.86-1.73)	TA	-2.73(-3.86--1.60)
0.32(-1.07-1.52)	0.03(-2.02-1.98)	-3.22(-5.71--0.78)	-0.23(-1.87-1.28)	-0.65(-1.96-0.55)	LIA

OOA: oral opioid analgesic; IOA: intravenous opioid analgesic; NOA: non-opioid analgesic; TA: topical anaesthetic; LIA: locally injected anaesthetic; HSG: hysterosalpingography; VAS: visual analogue score.

### VAS at 30 minutes or more after HSG

**Fig. 3:**
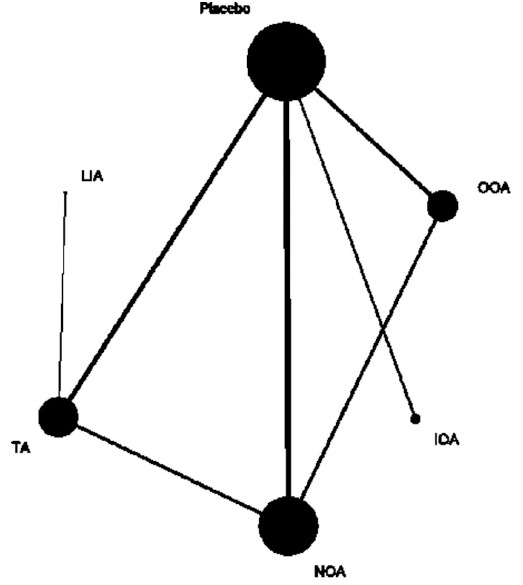
Network of treatment comparisons for VAS more than 30 minutes after HSG. The size of the node corresponds to the total sample size of treatments. Directly comparable treatments are linked with a line, the thickness of which represents the number of trials that were compared. OOA: oral opioid analgesic; IOA: intravenous opioid analgesic; NOA: non-opioid analgesic; TA: topical anaesthetic; LIA: locally injected anaesthetic; HSG: hysterosalpingography; VAS: visual analogue score.

Eleven trials were included in the network meta-analysis to evaluate this outcome. The VAS comparisons network at 30 minutes or more after HSG is shown in Fig. 3. Table-IV provides a hierarchy of effects based on VAS score at 30 minutes or more after HSG. The direct and indirect comparisons indicated that IOA resulted in excess but not statistically significant lower VAS pain score more than 30 minutes after HSG compared with the other groups. Based on SUCRA, IOA (0.7170) ranked as first, followed by LIA (0.6052), OOA (0.5862), TA (0.5102), NOA (0.4826) and placebo (0.0988).

**Table-IV:** Results for VAS pain score more than 30 minutes after HSG, from network meta-analysis (lower diagonal part) and pairwise meta-analysis (upper diagonal part).

Placebo	0.99(0.23-1.75)	1.80(0.78-2.82)	0.83(-0.20-1.86)	1.38(-0.68-3.44)	NA
1.23(-0.8-3.26)	OOA	NA	-0.60(-1.56-0.36	NA	NA
1.71(-1.11-4.23)	0.49(-3.14-3.73)	IOA	NA	NA	NA
0.95(-0.23-2.07)	-0.28(-2.38-1.83)	-0.77(-3.77-2.65)	NOA	-0.58(0.01-1.17)	NA
1.03(-0.6-2.83)	-0.19(-2.54-2.48)	-0.68(-3.82-2.74)	0.09(-1.44-1.64)	TA	0.31(-0.87-1.49)
1.41(-2.05-4.76)	0.19(-3.82-4.27)	-0.3(-4.49-4.31)	0.47(-2.82-3.76)	0.38(-2.63-3.31)	LIA

OOA: oral opioid analgesic; IOA: intravenous opioid analgesic; NOA: non-opioid analgesic; TA: topical anaesthetic; LIA: locally injected anaesthetic; HSG: hysterosalpingography; VAS: visual analogue score.

### Inconsistency and sensitivity analysis

The results obtained from the pairwise meta-analysis closely matched those of the network meta-analysis in the result of VAS during HSG, but inconsistencies were found when the Higgins model was used (Chi squared = 12.69, *P* = 0.0129). We also found that the inconsistencies derived from the comparison between placebo, TA and LIA. As regard VAS at 30 minutes or more after HSG, the results obtained from the pairwise meta-analysis closely matched those of the network meta-analysis and no inconsistencies were identified in the network meta-analysis when using the Higgins model (Chi squared = 0.78, *P* = 0.6781). The sensitivity analysis was performed by comparing the results of different effect model (random-effect and fixed-effect model). The results of random-effect model (pD=20.9 and DIC=23.3) were similar to the fixed-effect model (pD=15.9 and DIC=67.7).

## DISCUSSION

The network meta-analysis provides a hierarchy of effects due to the use of different pain killers, based on VAS pain score during HSG and VAS pain score at 30 minutes or more after HSG, which has advantages in the comparison with traditional pairwise meta-analyses.^8^

Our work presents the following strengths: (1) This network meta-analysis was conducted with a common method and was designed to allow a reproducible research selection and inclusion; (2), Broad and extensive search strategy were used to reduce the possibilities of publication bias; (3) The study overcame the main limitation of traditional pairwise meta-analysis by combining direct and indirect evidence of the treatments efficacy; (4) the posterior probabilities and SUCRA outcomes were used to rank the subtle differences among the six treatments.

However, this analysis has also some limitations. First, VAS results were inconsistent during HSG, which might reduce the significance of the conclusions. Second, only five trials report an adequate blinding process, it may have introduced performance bias and detection bias, in which the assessors are likely to have preferentially attributed injury occurrence to the control group. Finally, some included studies did not provide sufficient outcome data or report the information in a bar chart, for example, the standard deviation or the mean difference. Thus, to overcome the insufficient outcome data, some statistical methods were used, based on the data or bar chart supplied, as shown in this study.

Ahmad et al in 2007 conducted a Cochrane review including 12 randomized control trials, and they found that eight studies showed no difference in pain relief between patients under analgesics and those under placebo, while the remaining four studies showed a beneficial effect of analgesics when compared to placebos.^29^ Ahmad et al updated their review in 2015 and reported that only topical anesthetics and intravenous opioids were beneficial in reducing pain during the procedure. In addition, they did not found sufficient evidences regarding other drug treatments in reducing pain during HSG.^8^ Our network meta-analysis showed that IOA got excess but not statistically significant lower VAS pain score during HSG or more than 30 minutes after HSG compared with the other groups. We also found that OOA resulted in excess but not statistically significant higher VAS pain score during HSG compared with the other groups except placebo group. However, we also use the SUCRA and posterior probabilities of outcomes to distinguish the subtle differences among six treatments. For resulting in lower VAS pain score during HSG, the rank on treatments was: IOA, TA, NOA, LIA, OOA and placebo; For resulting in lower VAS pain score more than 30 minutes after HSG, the rank on treatments was: IOA, LIA, OOA, TA, NOA and placebo. However, as well as Ahmad^8^ insufficient evidence were found to lead to any conclusion regarding adverse effects.

## CONCLUSIONS

This new Bayesian network meta-analysis of data from randomized controlled trials demonstrated that IOA resulted in the highest probability to reduce the pain during HSG or at 30 minutes or more after HSG among the six interventions considered.
